# AID-FGS: Artificial intelligence-enabled diagnosis of female genital schistosomiasis: Preliminary findings

**DOI:** 10.1371/journal.pdig.0001255

**Published:** 2026-02-20

**Authors:** Akanksha Sharma, Tanmoy Dam, Sepo Mwangelwa, Chishiba Kabengele, William Kilembe, Bellington Vwalika, Mubiana Inambao, W. Evan Secor, Rachel Parker, Tyronza Skarkey, Susan Allen, Anant Madabhushi, Kristin M. Wall

**Affiliations:** 1 Wallace H. Coulter Department of Biomedical Engineering, Georgia Institute of Technology and Emory University, Atlanta, Georgia, United States of America; 2 Center for Family Health Research Zambia, Ndola, Zambia; 3 Center for Family Health Research Zambia, Lusaka, Zambia; 4 School of Medicine, University of Zambia, Lusaka, Zambia; 5 Division of Parasitic Diseases and Malaria, Centers for Disease Control and Prevention, Atlanta, Georgia, United States of America; 6 Rwanda Zambia HIV Research Group, Department of Pathology and Laboratory Medicine, School of Medicine and Hubert Department of Global Health, Rollins School of Public Health, Laney Graduate School, Emory University, Atlanta, Georgia, United States of America; 7 Department of Epidemiology, Rollins School of Public Health, Emory University, Atlanta, Georgia, United States of America; Indiana University Indianapolis, UNITED STATES OF AMERICA

## Abstract

Female genital schistosomiasis (FGS) is a sequela of infection with a waterborne parasite prevalent in sub-Saharan Africa and is associated with increased HIV risk. Diagnosis of FGS involves visual colposcopic identification of lesions on the cervix or vaginal walls. Previous studies have utilized digital image processing methods with statistical validation, and more recently, an artificial intelligence (AI)-based approach has also been explored. In this work, we sought to evaluate the performance of an AI model for identifying the presence of FGS from cervical photographs. Colposcopy images were obtained from 340 subjects in Zambia. Ground truth for presence or absence of FGS was determined by trained expert human examiners using visual assessment of images. Examiners also provided a FGS severity score between 0–8 for each image based on the number of lesions and the cervical quadrants affected, where 8 denotes highest severity and 0 denotes no FGS. The images were pre-processed with specular reflection artifact removal and image cropping to focus on the regions corresponding to the cervix and the transformation zone. The preprocessed dataset was randomly divided into training (FGS = 71, no FGS = 71) and testing (FGS = 21, no FGS = 177) cohorts. Image representations in the latent space were obtained using an ensemble of pre-trained machine learning models to further classify the image into FGS and no FGS. The best performance in the testing dataset was obtained at subject-level with area under the curve (AUC) =0.70 (95% Confidence interval: 0.58 - 0.82), Specificity = 0.68, and Sensitivity = 0.71, against the ground truth. Subjects with higher FGS severity scores (between 5–8) had high prediction rate by the machine classifier compared to those with lower severity scores (between 1–4). Machine learning shows promise in detecting FGS from limited colposcopy images. Early, accurate diagnosis may enhance reproductive health, and reduce HIV transmission risks, safeguarding maternal and child health.

## Author summary

Female genital schistosomiasis is a disease caused by a waterborne parasite *Schistosoma haematobium*, affecting millions of women in sub-Saharan Africa. It can lead to serious health problems and increases the risk of contracting HIV. Diagnosing this condition currently depends on a detailed visual exam of the cervix, performed by highly trained specialists. But these experts are scarce, and even among them, there can be disagreement when identifying the disease. In our study, we explored whether a computer-based system could help solve this problem. We used photos taken during medical exams to recognize patterns linked to the disease. This type of learning based on patterns in images is similar to how humans learn to recognize faces or objects. We found that the computer model was able to identify the disease with a level of accuracy that is encouraging, especially when the disease was more severe. This shows that technology can play a meaningful role in bringing better healthcare to places where expert diagnosis isn’t always available. Our hope is that this approach will lead to quicker, more reliable detection, helping protect the health of women and reduce their risk of HIV.

## Introduction

Infection with *Schistosoma haematobium*, a parasitic worm found primarily in sub-Saharan Africa, can result from direct contact with contaminated fresh water [1]. Infection can cause female genital schistosomiasis (FGS) which affects an estimated 56 million women and girls in sub-Saharan Africa and is one of the most neglected tropical diseases globally [1]. In endemic countries, women and girls often encounter *S. haematobium*-contaminated water during daily chores and activities [1]. Schistosomiasis infection in humans begins when larval forms of the parasite, released by infected freshwater snails, penetrate the skin during contact with contaminated water. Transmission is sustained when infected individuals release parasite eggs into freshwater through their urine or feces. These eggs hatch in the water, continuing the parasite’s lifecycle. Once inside the human body, the larvae mature into adult schistosomes that reside in blood vessels. Female worms release eggs, some of which exit the body to infect new water sources, while others become lodged in tissues, triggering immune responses and causing progressive organ damage [2].

FGS is characterized by lesions on the reproductive organs including the cervix which may cause reproductive organ damage, subfertility, pregnancy complications, lost productivity, stigma [3], cervical dysplasia [4,5], and increased HIV risk [6,7].

Female genital schistosomiasis (FGS) remains a largely neglected manifestation of schistosomiasis that is chronically underreported, misdiagnosed, and untreated by the global health community [1,8].The current clinical standard for FGS diagnosis is via visual assessment of the cervix and vaginal walls by trained clinicians either during colposcopy examination or from images of the cervix taken during colposcopy. Clinicians inspect the cervix and vaginal walls for four characteristic FGS lesions: grainy sandy patches, homogenous yellow sandy patches, abnormal blood vessels, or rubbery papules [9]. Challenges with this method in low and middle income countries (LMIC) include a dearth of providers trained to identify FGS, subjective and differing assessments among providers, and limited colposcopy equipment [8]. FGS shares symptoms with sexually transmitted infections (STIs) which can contribute to its misdiagnosis [9] and associated stigma [10,11]. As a result, for women living in *S. haematobium* endemic areas*,* FGS remains highly prevalent and under-diagnosed [1].

**Fig 1:**
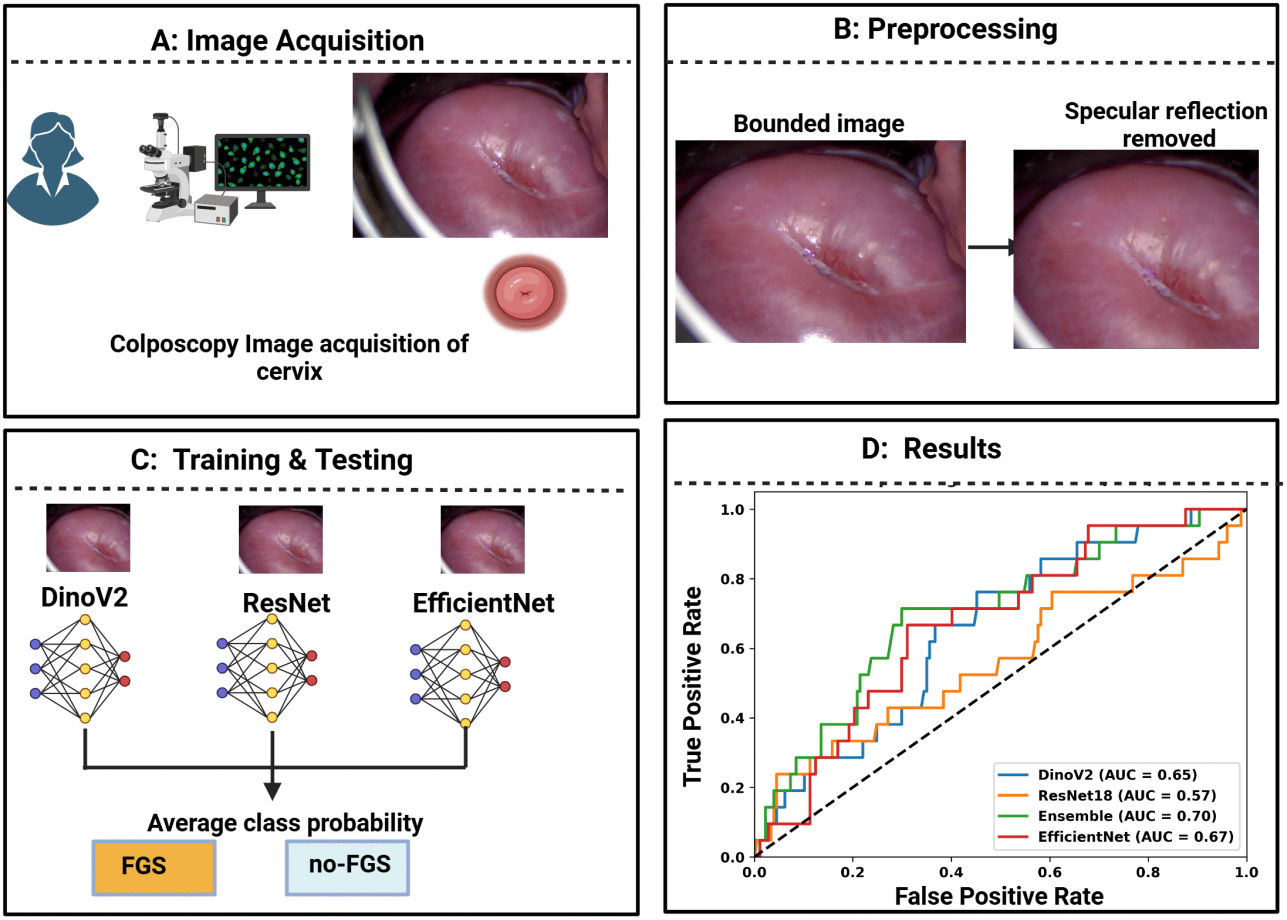
Overview diagram for the development of the Artificial Intelligence/Machine Learning (AI/ML) tool. **A)** Colposcopy image acquisition. **B)** Images were enclosed within a rectangular bounding box to remove unnecessary details and include the cervix ostium and transformation zone. The bounded images were processed to remove specular reflections. **C)** The ensembled model comprising of pretrained DinoV2, EfficientNet and ResNet were fine-tuned and tested with randomly divided training and test set. **D)** Results were computed using area under the curve (AUC) sensitivity, specificity, accuracy and F1 score.

Advancements in FGS diagnostic methods are urgently needed. Limited literature in this domain includes the use of computerized cervical image analysis approaches including colorimetric analysis [12,13], characterization of blood vessels [14], use of a grid to validate cervical lesion proportion [15] and the recent work [16] using Deep learning model YOLO. We undertook this study to evaluate the potential of deep learning-based AI methods to identify FGS using colposcopy images from 340 study participants (also referred to as subjects) from Zambia. The ground truth for the presence of FGS was established by expert obstetrician/gynecologist (OB/GYNs) using visual assessment as per the recommended clinical standard [9]. We also assessed the performance of the AI method (AID-FGS) with respect to the severity scores as well as the impact of image harmonization methods on the AI model’s performance. The methodology for development of the AI/Machine Learning (ML) tool included data acquisition, preprocessing by enclosing the image in a bounding box followed by specular reflection removal, learning the optimal feature representation, and classification to identify images with FGS. Fig 1 illustrates the block diagram of the methodology and the study design to develop the AI/ML algorithm.

## Materials and methods

### Study design and sampling

This is a cross-sectional study, nested within a prospective cohort. Members of the cross-sectional study were recruited from the cohort through convenience sampling. Center for Family Health Research Zambia (CFHRZ) nurse counselors approached participants while waiting to attend their annual or quarterly cohort study visits at the Lusaka or Ndola CFHRZ research sites. Women were provided with a description of this study, and those who were interested and provided written informed consent were enrolled by a CFHRZ nurse counselor.

### Ethics approval and consent to participate

This study was approved by the Institutional Review Board of Emory University and the University of Zambia Biomedical Research Committee. Participants provided written informed consent.

### Participant selection

From March 2020 to December 2021, we recruited 499 women from an existing prospective cohort of women at high risk for HIV in two large urban areas in Zambia (Lusaka and Ndola) [17]. Women in the cohort were either female sex workers (FSW) or single mothers who were at least 18 years of age. Women were referred to the CFHRZ research sites after community outreach at sex worker hotspots or post-natal clinics, respectively.

### Survey procedures

At CFHRZ research sites, participants completed baseline surveys in the local language (Nyanja in Lusaka and Bemba in Ndola) to assess demographics; reproductive, gynecological, and urinary history and symptoms; and potential environmental exposures to *S. haematobium*.

### Clinical procedures

Colposcopic exams were conducted on non-menstruating women. First, an autoclaved bivalve speculum was inserted, and genital examination assessed inflammation, contact bleeding, discharge, ulceration, and adenopathy. Then, the cervix was cleared of any discharge; endocervical and vaginal swabs were collected; and finally, a Bovie Colpo-Master CS-105LEDI Swing Arm Colposcope equipped with Continuous Zoom (zoom ratio 1:6.7 (0.67x-4.5x) and magnification 3.9x-27x) was used to take images of the cervix. Images were also taken both before (PRE images) and after a cervical wash with acetic acid (POST images) for visual inspection with an acetic acid (VIA) test. Our expert OB/GYN trained four CFHRZ research doctors and nurses to perform these procedures. Additionally, women provided urine samples for hematuria testing and urine filtration for the detection of *S. haematobium* eggs by trained laboratory technicians. Participants were also tested for gonorrhea, chlamydia, acute HIV infection, and high-risk human papillomavirus (hrHPV) by PCR; trichomoniasis, candida, bacterial vaginosis by microscopy; syphilis by Rapid Plasma Reagin (RPR); and HIV by rapid test. All testing was conducted by trained laboratory technicians as previously described [17].

### Clinical FGS diagnosis (ground truth)

Images taken during colposcopy were downloaded onto a computer for storage. One OB/GYN independently reviewed images applying a standard FGS case definition (i.e., presence of any FGS indicator: grainy sandy patches, homogenous yellow sandy patches, abnormal blood vessels, or rubbery papules) [9]. Each image was reviewed by one OB/GYN (either MI or BV; both are experts in FGS identification and the latter is a co-author of the World Health Organization FGS Pocket Atlas) [9]. The reviewer recorded whether there was the presence of any of the above listed indicators of FGS and where each indicator was located by cervical quadrant. A severity score of 0–8 was then assigned to each participant based on the total number of FGS indicators observed and the number of cervical quadrants involved [18]. Score 0 represents no FGS while 8 represents the highest FGS severity. A higher severity score would indicate presence of either more infected quadrants of the cervix and/or more FGS lesion types. For example, a score of 3 might reflect a case where the infection is spreading but not yet involving the entire cervix. A score of 4 indicates a more advanced or aggressive presentation, either due to broader anatomical involvement or more pronounced diagnostic features. As there are no established severity metrics for FGS, we derived this severity score and previously reported that increased severity was associated with decreased lesion resolution post-treatment [18], indicating the clinical utility of the derived severity score. To reduce bias, gynecological exam, laboratory, and survey findings were unknown to the OB/GYN at the time of image review.

### Treatments and referrals

Women with any FGS indicator, egg excretion, or hematuria were treated for free at CFHRZ research sites with praziquantel (40 mg/kg). Women diagnosed with STIs or vaginal dysbioses were treated for free at CFHRZ per Zambian National Guidelines [19] Women who were HIV, VIA, or hrHPV positive were referred for care to a local government health facility.

### Data collection and management

Survey data were collected on tablets using SurveyCTO (Dobility, Inc., v2.81.4). Clinical and laboratory data were collected on paper forms and later entered into SurveyCTO. Data were imported weekly from SurveyCTO into MS Access for long-term storage, quality control, and cleaning.

### Analysis dataset

**Fig 2:**
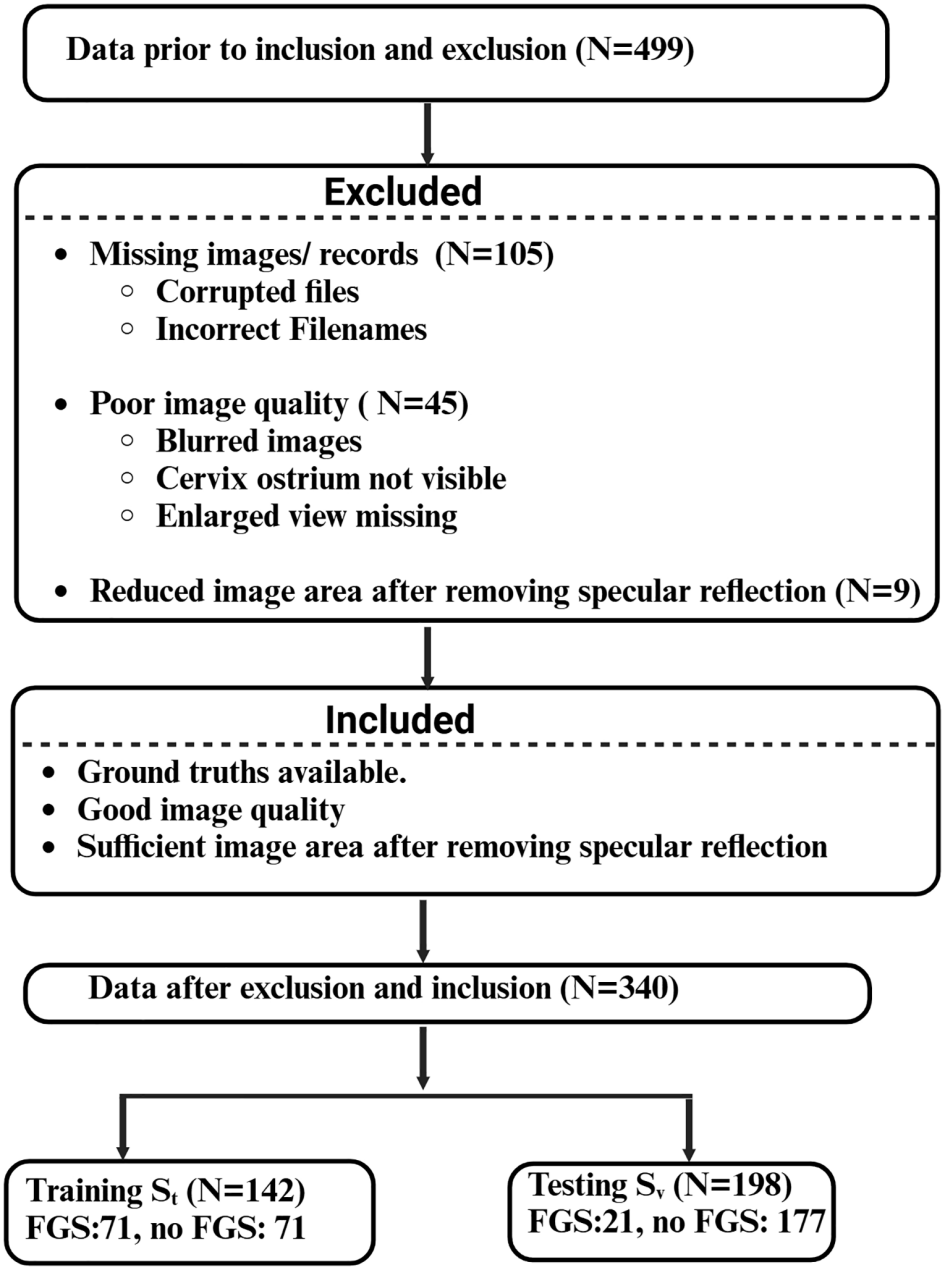
Diagram for inclusion and exclusion of participants for Artificial Intelligence/Machine Learning (AI/ML) tool development. Specular reflection refers to a prominent artifact in colposcopy images that hinders the analysis of lesions.

Fig 2 shows the block diagram for the inclusion and exclusion of subjects into the AI/ML analytic dataset. In total, 499 women were recruited for study. Among these women, 105 had missing images. Exclusion criterion involved blurred images, cervix ostrium not visible, enlarged view of cervix missing. The inclusion criterion involved ground truth available, good image quality and lesser reduction in image area after removing specular reflections. Following these, we excluded 54 participants and included 340 participants in the study, resulting in 92 women with FGS and 248 women who did not have FGS (referred to as no FGS).

### Preprocessing

**Fig 3:**
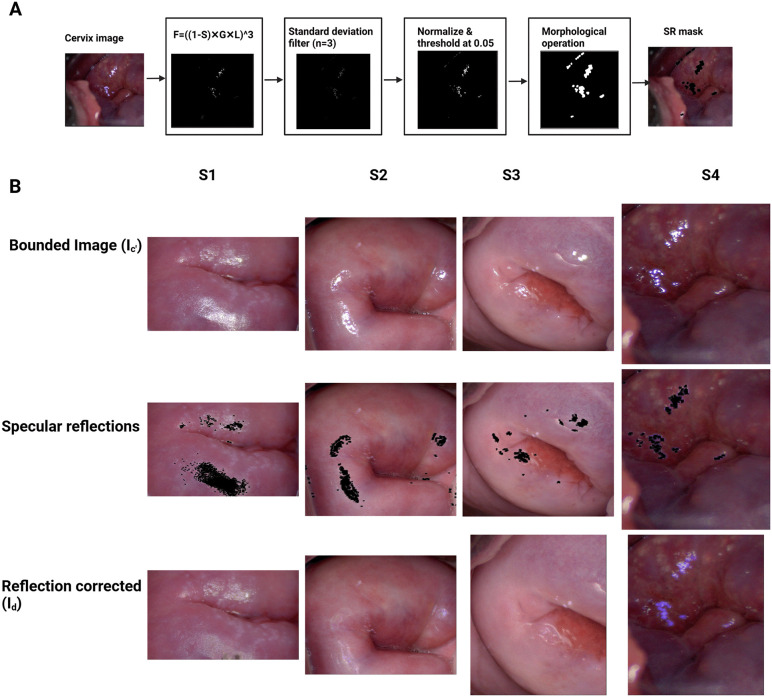
Diagram for detecting the specular reflections (SR) on colposcopy images. **(A)** The S, G, and L components for computing F, correspond to saturation **(S)**, green (G) and luminance (L) channels, obtained from HSV, RGB and CIE-Lab color spaces respectively. **(B)** SR removal for four FGS positive women, $S_{i}\;(\;i=\text{1},\text{2},\text{3},\text{4})$. The first row shows the bounded image, the second row shows the highlights (in black pixels) detected using method defined in **(A)**, and the third row shows the images with SR removed using diffusion modeling.

Colposcopy images often capture unnecessary features, like the speculum. To exclude these details and computation in the images, colposcopy images ($I_{c}$) (3072 × 1728 pixels) were cropped manually into a rectangular bounding box $\;(I_{c^{\prime }})$ using the Label Studio software [20,21]. Specular reflections (SR) or highlights are strong artifacts that sometimes accompany cervix images. SR appear as bright white areas on cervix images, resulting from light reflecting off the cervix’s wet surface [22]. These highlights disrupt the content analysis of the surrounding region as well as lesions on those sites, necessitating the removal of these artifacts. SR were identified using the method described in a publication [23], except that the threshold after filtering was adjusted to 0.05 grayscale value as per our dataset. Fig 3(A) illustrates the method used for detecting highlights on our dataset. The pixels corresponding to the highlighted regions were filled using a pre-trained diffusion model [24,25]. The diffusion models are generative models used in image synthesis and denoising that produce realistic high-quality images [24]. To retain the original image resolution, a tile-based approach was used to fill the SR regions in $\left(I_{c^{\prime }}\right)$ to generate $I_{d}$. $I_{c^{\prime }}$ was divided into tiles with dimensions of 512 × 512 pixels. The tiles corrected for highlights were then combined to obtain $I_{d}.$ Sample images from our dataset are illustrated in Fig 3(B).

### Machine learning

The newly presented AID-FGS machine learning model to classify FGS and no FGS comprises an ensemble of pre-trained models including DinoV2 [26], EfficientNetB0 [27] and ResNet18 [28]. DinoV2 was selected as a representative transformer-based approach because it learns robust visual representations through self-supervised learning without requiring large labeled datasets [26]. DinoV2 has demonstrated strong transfer learning capabilities across diverse visual recognition tasks, including medical imaging applications [29,30]. EfficientNetB0 was chosen for its optimal balance between accuracy and computational efficiency. EfficientNetB0 offers a practical advantage over larger, more computationally demanding models and has shown its potential in transfer learning on medical images [27,31]. A recent survey on cervical cancer algorithms eligible for FGS detection identified ResNet as one of the most frequently used deep learning models in this domain [8], supporting its relevance for our application. We employed transfer learning [32] to fine tune and optimize the aforementioned models. Transfer learning refers to an approach where we leverage the knowledge gained from the source domain to improve learning efficiency and performance on the target domain [32]. Details on Transfer Learning approach used in AID-FGS are described in Section A in S1 Text. Training specific details and parameters are listed in Section B in S1 Text. Along with the ensemble model, DinoV2, EfficientNetB0, and ResNet18 were also used in the same paradigm to evaluate the efficacy of each model for FGS detection. Integrated gradient (IG) maps [33] were obtained to understand the learning patterns of the model. Each model resulted in a heatmap using the gradients of the model’s output with respect to input, in turn highlighting which features of the input were most influential in the model’s overall decision. T-distributed stochastic neighbor embedding (t-SNE) is a dimensionality reduction method that helps to visualize high-dimensional data [34]. It creates a plot in 2D/3D space where essential features are used to show underlying patterns in the data. In our study, t-SNE was used to plot high-dimensional embeddings obtained from ML models for FGS positive participants in $S_{v}$ to observe their grouping with respect to severity scores.

### AI methods for detecting cervical cancer

A recent extensive review [8] explored whether AI algorithms designed for cervical cancer/dysplasia detection using colposcopy images could be applied to FGS detection. The methodology described in this study prompted us to explore handcrafted features described by Xu et al. [35] for FGS detection. For each image, we extracted three complementary pyramid features: Pyramid histogram in L*A*B* color space (PLAB), Pyramid Histogram of Oriented Gradients (PHOG), and Pyramid histogram of Local Binary Patterns (PLBP). Support vector machine was used along with linear and radial basis function (rbf) kernel to evaluate the performance of handcrafted features using uncorrelated features. The collinear features were identified with a Spearman correlation coefficient greater than or equal to 0.6. The performance of these features was also evaluated using a similar holdout test set.

### Statistical analysis

Due to the limited number of FGS positive participants in the dataset, we divided the samples into a balanced training set ($S_{t}$, FGS = 71, no FGS = 71) and an imbalanced testing set ($S_{v}$, FGS = 21, no FGS = 177), reflecting real-world clinical scenarios [36]. Sensitivity, specificity, accuracy, area under the curve (AUC), 95% confidence interval (CI) of AUC, and F1 score were computed to assess the performance of each of the models. The gold standard diagnosis (true label) was taken from the expert OB/GYN. The sensitivity and specificity were computed using the following formula for the predicted labels by the deep learning model.


$$\text{Sensitivity}=\frac{\text{True Positive}}{\text{True Positive}+\text{False Negatives}}$$

$$\text{ Specificity}=\frac{\text{True Negatives}}{\text{True Negatives}+\text{False Positive}}$$

The F1 score refers to the harmonic mean of precision and recall and is a suitable metric to estimate performance for an imbalanced class distribution [37]. The interpretation of AUC values is defined as AUC = 0.5: no discrimination, 0.5 < AUC < 0.7: poor discrimination, 0.7 ≤ AUC < 0.8: acceptable discrimination, 0.8 ≤ AUC < 0.9 excellent discrimination, AUC ≥ 0.9 outstanding discrimination [38]. The interpretation of sensitivity and specificity is similar to accuracy, the higher the better. We continuously evaluated results on $S_{v}$ during the training phase of the model. The maximum F1 score attained across all training iterations on $S_{v}$ subject to the constraint that the sensitivity and specificity were greater than 0.6 was considered as the criterion for finalizing the trained model. In case the model fails to attain a cut-off of 0.6 for sensitivity and specificity, the highest F1 score was considered for finalizing the trained model. De Long’s method [39] was used for computing the CI of the AUC. Python’s Scikit library [40] and PyTorch [41] were used for the implementation of the algorithm.

## Results

### Patient characteristics

The distribution of the four typical FGS lesions is described in Table 1. Presence of abnormal blood vessel was the most prominent FGS lesion among participants, while rubbery papules were the least frequent lesion type. Table 2 shows the baseline characteristics for demographics and clinical examination for subjects.

**Table 1:** Female genital schistosomiasis (FGS) lesion distribution.

Variable of interest	Total (N = 340)		Female sex workers (N = 230)		Single mothers (N = 110)	
	N	%	N	%	N	%
**Any FGS indicator**						
Positive	92	27.1%	61	26.5%	31	28.2%
Negative	248	72.9%	169	73.5%	79	71.8%
**Grainy sandy patches**						
Positive	16	4.7%	9	3.9%	7	6.4%
Negative	324	95.3%	221	96.1%	103	93.6%
**Homogenous yellow sandy patches**						
Positive	42	12.4%	27	11.7%	15	13.6%
Negative	298	87.6%	203	88.3%	95	86.4%
**Abnormal blood vessels**						
Positive	62	18.2%	39	17.0%	23	20.9%
Negative	278	81.8%	191	83.0%	87	79.1%
**Rubbery papules**						
Positive	6	1.8%	5	2.2%	1	0.9%
Negative	334	98.2%	225	97.8%	109	99.1%
***S. haematobium* Ova from urine**						
Positive	20	6.1%	11	5.0%	9	8.3%
Negative	309	93.9%	210	95.0%	99	91.7%
**Age**	27.2	4.9	27.1	4.8	27.4	5.2

**Table 2:** Baseline characteristics of subject with respect to demographics and clinical examination.

	N missing	Total (N = 340)		Diagnosed with FGS (N = 92)		Not diagnosed with FGS (N = 248)		p-value (2-tailed)
		N	%	N	%	N	%	
**DEMOGRAPHICS**								
**Age (median/IQR)**		26.0	6.0	27.0	4.0	26.0	6.0	0.1352
**City**								0.4639
Lusaka		170	50.0%	43	46.7%	127	51.2%	
Ndola		170	50.0%	49	53.3%	121	48.8%	
**Province of Birth**								0.1267
Copperbelt		161	47.4%	42	45.7%	119	48.0%	
Lusaka		107	31.5%	24	26.1%	83	33.5%	
Other		72	21.2%	26	28.3%	46	18.5%	
**REPRODUCTIVE HEALTH SYMPTOMS**								
**Do you sometimes have to rush to the bathroom because you get a sudden, strong need to urinate? (Binary)**								0.2103
Yes		80	23.5%	26	28.3%	54	21.8%	
No		260	76.5%	66	71.7%	194	78.2%	
**History of bloody urine**								1.0000
Yes		5	1.5%	1	1.1%	4	1.6%	
No		335	98.5%	91	98.9%	244	98.4%	
**History of genital ulcer**								0.6635
Yes		6	1.8%	2	2.2%	4	1.6%	
No		334	98.2%	90	97.8%	244	98.4%	
**History of genital warts/growths**								0.3935
Yes		7	2.1%	3	3.3%	4	1.6%	
No		333	97.9%	89	96.7%	244	98.4%	
**History of contact pain during intercourse**								0.0403
Yes		19	5.6%	9	9.8%	10	4.0%	
No		321	94.4%	83	90.2%	238	96.0%	
**Are you having any reproductive health problems that you would like to talk to the nurse/doctor about today?**								0.6878
Yes		8	2.4%	1	1.1%	7	2.8%	
No		332	97.6%	91	98.9%	241	97.2%	
**Cystitis/dysuria? (Binary)**								0.6635
Yes		6	1.8%	2	2.2%	4	1.6%	
No		334	98.2%	90	97.8%	244	98.4%	
**Vaginal itching? (Binary)**								0.5262
Yes		13	3.8%	2	2.2%	11	4.4%	
No		327	96.2%	90	97.8%	237	95.6%	
**Abnormal Vaginal discharge (Binary)**								0.7074
Yes		9	2.6%	3	3.3%	6	2.4%	
No		331	97.4%	89	96.7%	242	97.6%	
**Dyspareunia (painful intercourse) (Binary)**								0.6878
Yes		8	2.4%	1	1.1%	7	2.8%	
No		332	97.6%	91	98.9%	241	97.2%	
**Lower abdominal pain**								0.7337
Yes		10	2.9%	3	3.3%	7	2.8%	
No		330	97.1%	89	96.7%	241	97.2%	
**Acute genital ulcer**								0.2706
Yes		1	0.3%	1	1.1%	0	0.0%	
No		339	99.7%	91	98.9%	248	100.0%	
**Chronic/ Recurrent genital ulcer**								0.2964
Yes		4	1.2%	2	2.2%	2	0.8%	
No		336	98.8%	90	97.8%	246	99.2%	
**Unpleasant vaginal odor/malodorous discharge**								1.0000
Yes		1	0.3%	0	0.0%	1	0.4%	
No		339	99.7%	92	100.0%	247	99.6%	
**Pelvic/back pain**								0.1760
Yes		11	3.2%	5	5.4%	6	2.4%	
No		329	96.8%	87	94.6%	242	97.6%	
**Chronic/recurrent genital warts/growths (small bump, cluster of bumps, or stemlike protrusions) on your genitalia (vulva, vagina, or anus)**								1.0000
Yes		2	0.6%	0	0.0%	2	0.8%	
No		338	99.4%	92	100.0%	246	99.2%	
**GENEXPERT STI RESULTS**								
**CT**	2							0.6720
Yes		26	7.7%	8	8.7%	18	7.3%	
No		312	92.3%	84	91.3%	228	92.7%	
**NG**	2							0.7170
Yes		21	6.2%	5	5.4%	16	6.5%	
No		317	93.8%	87	94.6%	230	93.5%	
**Any HPV (HPV16, HPV18/45, High-risk HPV)**	13							0.9805
Yes		149	45.6%	40	45.5%	109	45.6%	
No		178	54.4%	48	54.5%	130	54.4%	
**LABORATORY/MICROSCOPY DATA**								
**RPR**	63							0.8699
Yes		30	10.8%	7	10.3%	23	11.0%	
No		247	89.2%	61	89.7%	186	89.0%	
**Sperm**	25							0.4534
Yes		9	2.9%	1	1.2%	8	3.5%	
No		306	97.1%	83	98.8%	223	96.5%	
**Trichomonas**	22							0.4024
Yes		17	5.3%	6	7.1%	11	4.7%	
No		301	94.7%	78	92.9%	223	95.3%	
**Candida**	12							0.6858
Yes		8	2.4%	1	1.2%	7	2.9%	
No		320	97.6%	85	98.8%	235	97.1%	
**Bacterial Vaginosis (BV)**	12							0.2472
Yes		66	20.1%	21	24.4%	45	18.6%	
No		262	79.9%	65	75.6%	197	81.4%	
**GYNECOLOGICAL EXAM**								
**External genitalia**								
Inguinal adenopathy > 1 cm 2 cm unilateral	1							1.0000
Yes		1	0.3%	0	0.0%	1	0.4%	
No		338	99.7%	92	100.0%	246	99.6%	
Inguinal adenopathy > 1 cm 2 cm bilateral	1							0.4697
Yes		2	0.6%	1	1.1%	1	0.4%	
No		337	99.4%	91	98.9%	246	99.6%	
Inflammation	1							---
Yes		0	0.0%	0	0.0%	0	0.0%	
No		339	100.0%	92	100.0%	247	100.0%	
Ulceration	1							1.0000
Yes		1	0.3%	0	0.0%	1	0.4%	
No		338	99.7%	92	100.0%	246	99.6%	
Condyloma/ Warts	1							---
Yes		0	0.0%	0	0.0%	0	0.0%	
No		339	100.0%	92	100.0%	247	100.0%	
Tumors/nodules on the vulva/vagina	1							---
Yes		0	0.0%	0	0.0%	0	0.0%	
No		339	100.0%	92	100.0%	247	100.0%	
**Internal genitalia**								
Inflammation cervix/ Cervicitis								0.0202
Yes		23	6.8%	11	12.0%	12	4.8%	
No		317	93.2%	81	88.0%	236	95.2%	
Inflammation vagina								1.0000
Yes		1	0.3%	0	0.0%	1	0.4%	
No		339	99.7%	92	100.0%	247	99.6%	
Ulcer cervix								---
Yes		0	0.0%	0	0.0%	0	0.0%	
No		340	100.0%	92	100.0%	248	100.0%	
Ulcer vagina								---
Yes		0	0.0%	0	0.0%	0	0.0%	
No		340	100.0%	92	100.0%	248	100.0%	
Non-bloody Discharge/ Pus originating from cervix								0.0271
Yes		10	2.9%	6	6.5%	4	1.6%	
No		330	97.1%	86	93.5%	244	98.4%	
Non-bloody Discharge vagina								0.6558
Yes		40	11.8%	12	13.0%	28	11.3%	
No		300	88.2%	80	87.0%	220	88.7%	
Bloody Discharge/ Pus originating from cervix								0.4685
Yes		2	0.6%	1	1.1%	1	0.4%	
No		338	99.4%	91	98.9%	247	99.6%	
Bloody Discharge vagina								0.5660
Yes		3	0.9%	0	0.0%	3	1.2%	
No		337	99.1%	92	100.0%	245	98.8%	
Erosion or friability cervix								1.0000
Yes		7	2.1%	2	2.2%	5	2.0%	
No		333	97.9%	90	97.8%	243	98.0%	
Erosion or friability vagina								---
Yes		0	0.0%	0	0.0%	0	0.0%	
No		340	100.0%	92	100.0%	248	100.0%	
Non-menstrual bleeding cervix								1.0000
Yes		2	0.6%	0	0.0%	2	0.8%	
No		338	99.4%	92	100.0%	246	99.2%	
Non-menstrual bleeding vagina								---
Yes		0	0.0%	0	0.0%	0	0.0%	
No		340	100.0%	92	100.0%	248	100.0%	
Condyloma/ Warts cervix								---
Yes		0	0.0%	0	0.0%	0	0.0%	
No		340	100.0%	92	100.0%	248	100.0%	
Condyloma/ Warts vagina								0.4685
Yes		2	0.6%	1	1.1%	1	0.4%	
No		338	99.4%	91	98.9%	247	99.6%	
Tumors/nodules on the cervix	1							0.2714
Yes		1	0.3%	1	1.1%	0	0.0%	
No		338	99.7%	91	98.9%	247	100.0%	
Adnexal tenderness	3							---
Yes		0	0.0%	0	0.0%	0	0.0%	
No		337	100.0%	91	100.0%	246	100.0%	
Adnexal mass	4							---
Yes		0	0.0%	0	0.0%	0	0.0%	
No		336	100.0%	91	100.0%	245	100.0%	
VIA Positive	10							0.0005
Yes		89	27.0%	37	40.7%	52	21.8%	
No		241	73.0%	54	59.3%	187	78.2%	

**Note:** N refers to median and % refers to Inter-quartile range.

### Performance of ensemble model

**Fig 4:**
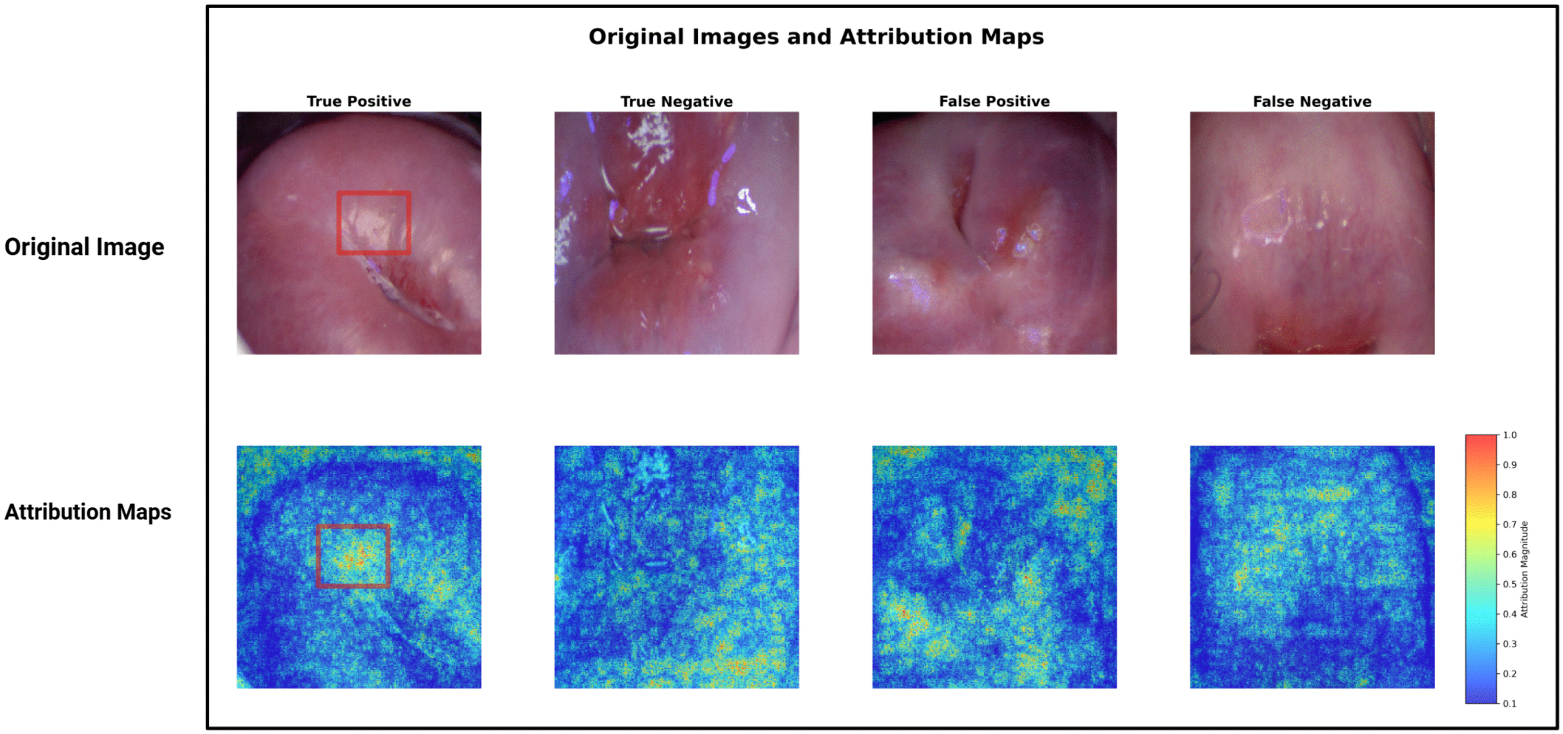
Integrated gradient maps and colposcopy images of True Positive (correctly classified female genital schistosomiasis (FGS), True Negative (correctly classified as no FGS), False Positive (misclassified as FGS), and False Negative (misclassified as no FGS) women. The red bounding box in True Positive column, shows a yellow sandy patch, a characteristic FGS lesion. The Attribution maps show high values of gradient in the corresponding region.

**Fig 5:**
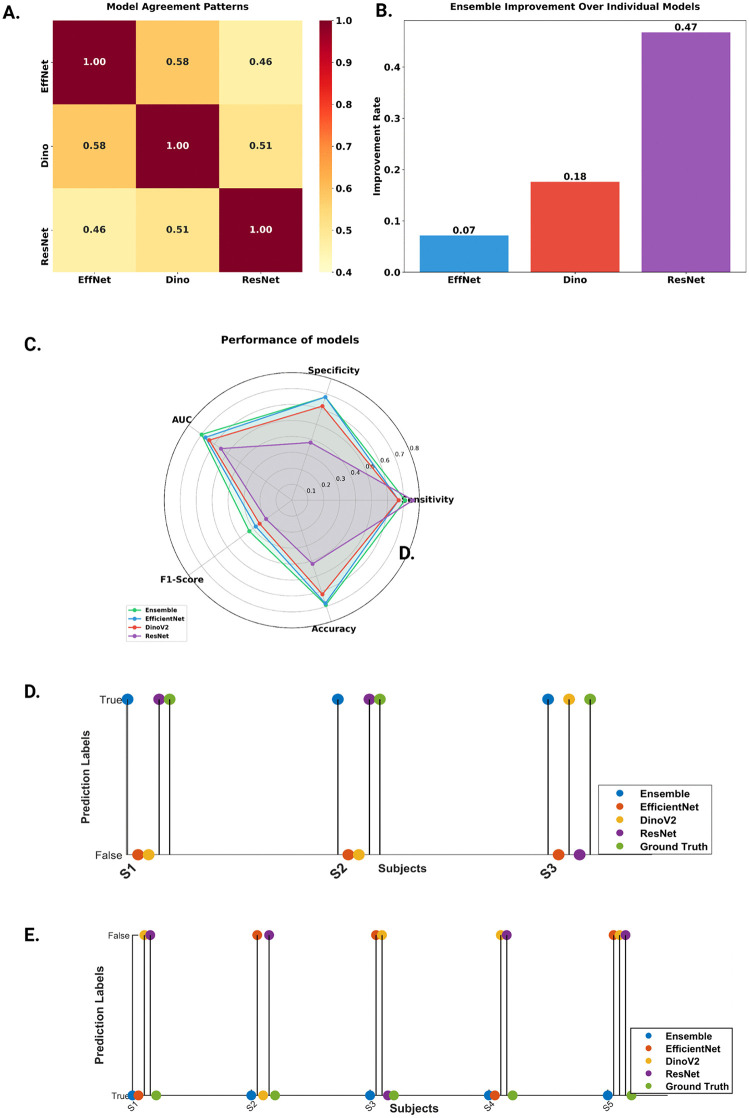
Rationale for model ensemble A: Heatmap for model agreement rate for three individual models B: Bar plot showing relative improvement of the ensemble model over the three individual models. **C**: Radar plot illustrating the performance characteristics of all the models **D:** Prediction of three FGS study participants with the different models compared to the obstetrician/gynecologist (OB/GYN) classifications (ground truth) **E:** Prediction of five women without FGS using different models along with ground truth.

Best performance at individual participant-level on $S_{v}$ was obtained as AUC = 0.70 (95% CI: 0.58 - 0.82), sensitivity = 0.71, specificity = 0.68, and F1 score = 0.33. The performance metrics on $S_{t}$ were AUC = 0.65 (95% CI: 0.56 - 0.74), sensitivity = 0.58, specificity = 0.72, and F1 = 0.62. Fig 4 shows the IG maps of correctly classified FGS and no FGS participants. A yellow patch is visible in the colposcopy image of the FGS subject (red bounding box in the image). The corresponding pixels in IG show high gradient values in the area of the lesion region, suggesting that the model is capturing the FGS attributes. The performance of other established deep learning models is shown in Table 3. The ROC curves of all the deep learning models are shown in Fig 1(D). The figure suggests that Ensemble models perform better than other models for lower thresholds of False Positive Rate. Fig 5(A) shows the agreement heatmap of models with each other. Fig 5(B) shows the improvement rate of the ensemble model over the individual models. Fig 5(C) shows the radar plot of all the metrics for each model. It can be observed that EfficientNet and Ensemble model have a comparable overlap for all the metrics, except F1 Score. Conversely, ResNet18 attains the highest sensitivity, but performs worse for the other metrics.

**Table 3:** Performance of different deep learning models.

Models	AUC* (95% CI)	Sensitivity	Specificity	F1 Score	Accuracy
**Ensemble**	**0.70** **(0.58–0.82)**	**0.71**	**0.68**	**0.33**	**0.69**
**DinoV2**	0.64 (0.53–0.76)	0.67	0.62	0.25	0.62
**EfficientNet**	0.67 (0.56–0.79)	0.67	0.68	0.28	0.68
**ResNet18**	0.55 (0.48–0.70)	0.76	0.38	0.20	0.42

* Area under curve.

### Impact of preprocessing

**Fig 6:**
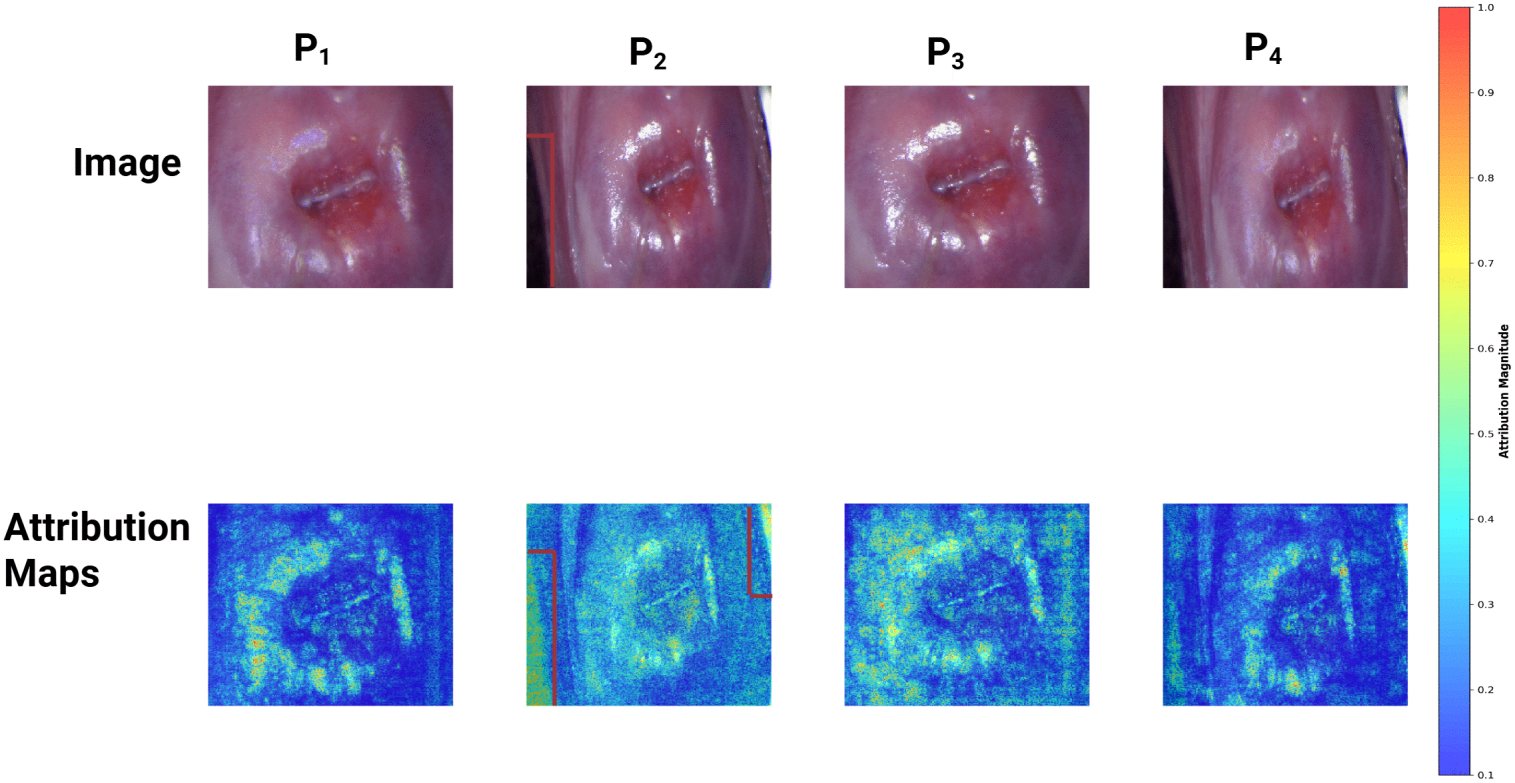
Image harmonization effect on ensemble model’s performance. The first row shows colposcopy images corresponding to four pre-processing cases for women with FGS. **P**_**1**_: Cropped + Inpainted, **P**_**2**_ Uncropped+ not-inpainted, **P**_**3**_ - Cropped+not-inpainted. **P4**-Uncropped+inpainted. The second row illustrates corresponding IG maps for the model. The red bounding box in panel P_2_ shows the higher values of gradients in background regions without any lesion signature. In panel P_1_, the model reveals improved gradient highlights in the cervix region, rather than focusing on regions of non-interest.

We performed an ablation study to analyze the effect of SR removal and cropping on the ensemble model’s performance. We evaluated four types of image pre-processing scenarios ($P_{i},\;i=\text{1},\text{2},\text{3},\text{4}$) on the dataset. $P_{\text{1}}$refers to preprocessing steps as used in AID-FGS (i.e., cropped images of the region of interest and the inpainted regions of SR). $P_{\text{2}}$ refers to no preprocessing (original images captured during colposcopy, i.e., uncropped and no inpainting to remove SR). $P_{\text{3}}$ refers to cropped images to the region of interest but not inpainted to remove SR. P4 refers to uncropped images but inpainted to remove SR. Table 4 shows the comparison of the performance measures for these approaches. The original images ($P_{\text{2}}$) fail to provide any signal to capture FGS. However, cropping the region of interest ($P_{\text{3}}$) provides relative improvement of 16.66% on AUC over $P_{\text{2}}$, but is still inferior to AID-FGS ($P_{\text{1}}$) because of the presence of artifacts. The lower performance of P4 compared to P1 confirms that cropping plays a pivotal role. Fig 6 shows the IG heatmap for a women with FGS using these four approaches, wherein we observe that the model focuses on the region of interest in AID-FGS (Panel 1) instead of the background region (speculum, wall lining) in Panel 2.

**Table 4:** Effect of image harmonization method’s on ensemble model’s performance.

Pre-processing	Cropped	Inpainted	AUC* (95% CI)	Sensitivity	Specificity	F1 Score	Accuracy
**P**_**1**_ **(AID-FGS)**	**Yes**	**Yes**	**0.70** **[0.58–0.82]**	**0.71**	**0.68**	**0.33**	**0.69**
P_2_	No	No	0.48 [0.33–0.63]	0.57	0.61	0.24	0.61
P_3_	Yes	No	0.56 [0.42–0.69]	0.67	0.58	0.26	0.59
P_4_	No	Yes	0.54 [0.40–0.67]	0.76	0.41	0.20	0.45

* Area under curve.

### Performance of ensembled model with respect to severity scores

**Fig 7:**
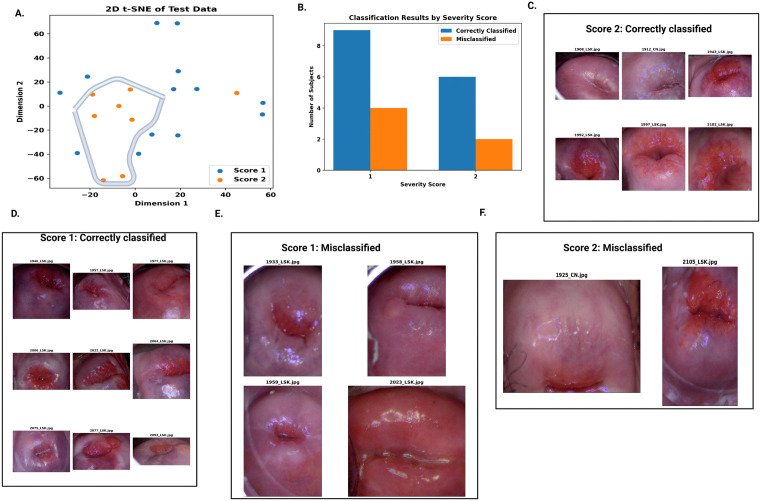
Severity score analysis of female genital schistosomiasis (FGS) in test data. **A.** t-SNE plot of embeddings of study participants in $S_{v}$ using Efficient Net model, grouped by severity scores. **B.** Bar graph of number of classified/misclassified women as per severity scores. **C.** Sample images of correctly classified women for severity score 2. **D.** Sample images of correctly classified women for severity score 1. **E.** Sample images of misclassified women for severity score 1. **F.** Sample images of women misclassified for severity score 2.

We converted the severity scores assigned by expert OB/GYN to dichotomous categories by grouping original scores of {1,2,3,4} as Score 1 and scores of {5,6,7,8} as Score 2. Additional detail on grouping of scores is described in Fig A in S1 Text. Fig 7(A) shows the t-SNE plot of embeddings from EfficientNet for FGS subjects in $S_{v}$, grouped by severity score. The subjects are grouped into clusters as per their scores. In Fig 7(B) we observe the bar plot of correctly classified and misclassified subjects with respect to scores. For the Score 1 grouping, the true prediction rate was 69.23%, while for Score 2 grouping, the true prediction rate was 75%. Fig 7(C-F)) illustrates the sample images of participants classified/misclassified as per the Score category.

### Comparison with AI methods for detecting cervical cancer

The handcrafted features resulted in a maximum AUC of 0.52 (95% CI: 0.38-0.63) with sensitivity and specificity of 0.42 and 0.58, respectively, using the rbf kernel. Handcrafted features performed at only chance-level accuracy for FGS detection. These features capture local color, gradient, and texture information of the cervix at different scales. However, the weak FGS characteristics in our dataset were not captured by them.

## Discussion

In this study, we present an AI-based model to diagnose FGS using images of the cervix taken during colposcopy. To the best of our knowledge, this is one of the few AI approaches to address the specific problem of FGS diagnosis using images of the cervix taken during colposcopy (AID-FGS). We examined the potential of both ensembled deep learning models as well as individual models to identify FGS. Among all approaches, transfer learning using the ensembled model demonstrated the best performance characteristics. For misclassified FGS subjects, the incorrect predictions could be driven by several factors including (1) the lesions are not visually prominent, (2) low illumination, including the field of view of the cervix is not enlarged and centered enough to capture the relevant features and (3) the SR corrected regions (with high activation values) are incorrectly interpreted as lesions. A previous study that attempted to develop and validate a measure for quantifying cervical lesion proportion in digital images of the cervix [15] found that one of the prominent reasons for disagreement between readers could be attributed to differences in the zoom level of the digital images resulting in different coverage of the cervix surface by the grid.

The impact of image harmonization methods on model’s performance demonstrate that removing artifacts and cropping images to their region of interest helps the model to better focus and capture relevant FGS signatures. However, in the future, the manual cropping of images (to focus on the cervix and transformation zone) could be automated to minimize manual intervention and make the pipeline more accessible for use by less experienced interventionalists. In contrast, the study by Zhu *et al.* [16] did not perform artifact removal or image cropping. Instead, their approach involved annotating bounding boxes around specific lesions. Thus, while both studies address the challenge of FGS detection, they employ distinct strategies. Our work emphasizes image-level classification, whereas Zhu *et al.* focused on lesion localization and detection.

New advanced deep learning models are being released with regular frequency. As a result, it is challenging to determine which models to use. We studied the performance of three individual established models as well as an ensemble of these models. None of the individual established models consistently provide correct predictions (Fig 5D and E). For example, for participant S5 (woman without FGS in Fig 5E), none of the individual models yielded accurate predictions. ResNet18 frequently predicted FGS in women who did not have FGS but accurately predicted FGS in women with the characteristic lesions. EfficientNet, which is designed to be more computationally efficient, often achieving better performance with fewer parameters and lower computational costs [27] performed better than DinoV2 and ResNet18, but still not as well as the ensemble model (Fig 5C). Regarding the ensemble design, our objective behind simple averaging was to maintain stability and reduce overfitting on a small dataset. We acknowledge that more sophisticated ensemble strategies, including optimized weighting schemes like attention and integration of diverse architectures such as Vision Transformers could further improve performance.

We also analyzed the performance of the ensembled model with respect to the severity score. Our findings indicate that FGS severity is a factor to consider in the classification performance. Higher grade lesions were easier to identify by machine learning models compared to lower grade lesions. However, further validation is required with additional studies.

Beyond FGS, colposcopy images have been extensively explored for cervical cancer screening. Study by Yuan et al. [42] used two colposcopy images corresponding to one acetic image and one iodine image along with age, HPV testing result, cytology result and type of transformation zone as the input to the deep learning model. The multimodal system attained sensitivity, specificity and accuracy of 85.38%, 82.62% and 84.10% respectively, with an AUC of 0.93 on dataset of 22396 images. Another study by Ouh et al [43] analyzed 9639 Tele-cervicography images, using deep learning based system for cervix detection as well as classification. In a multicenter retrospective study, their system attained sensitivity, specificity of 98%, 95%, respectively. Further, a recent review study by Lei Lu et al [44] concluded that AI systems achieved superior diagnostic accuracy compared to experienced colposcopists. By contrast, when we explored analysis techniques that attained satisfactory performance for identifying cervical dysplasia using acetowhite lesions [35] to our data, the results did not correspond well with ground truth FGS determinations. Comparing cervical cancer AI to FGS AI is essentially an “apples to oranges” comparison. Unlike cervical cancer, which benefits from decades of standardized, large-scale digital screening archives [8,45], FGS research faces significant challenges in data collection due to the lack of specialized training for providers and the hurdles of imaging in endemic, resource-limited settings [46]. Consequently, FGS datasets are inherently smaller [12,15]. The distinct pathophysiology of FGS lesions requires specialized models rather than the mere adaptation of cervical cancer algorithms, and our findings underscore the need for FGS-specific diagnostic frameworks.

Previously published FGS diagnostic techniques have been based on both clinical evaluations [47,48] as well as automated image processing methods using statistical validation. *S. haematobium* antigen detection [49], egg detection, and molecular diagnostic techniques such as PCR have been used to screen for *S. haematobium* from vaginal swabs [50–54]. However, these methods only give an indication of past or current infection with *S. haematobium* and not the presence of FGS lesions. Differences in age profiles, infection chronicity, and diagnostic modality (molecular vs. lesion/visual) introduce important limitations for interpreting our results and comparing across studies. Many young women may have active molecular evidence of infection (egg/antigen/DNA) but may not yet have developed the characteristic visual lesions or transformation zone changes captured in clinical/colposcopic inspections. For example, studies have found that cervicovaginal DNA or antigen markers can be positive in absence of visible lesions [45]. On the other hand, older women may have a lower prevalence of egg excretion (active infection) but higher prevalence of visual lesions (reflecting years of prior infection and tissue change), making comparisons across age groups non-straightforward. Visual methods capture morphological changes rather than direct parasite presence. They may under-detect early disease (in younger women) or over-estimate the risk of active disease in older women when lesions persist, but infection is resolved.

In a previous study [36], our research group explored a simple prediction tool using the demographics, risk factors, and symptom data like strong clinical indicators, visual inspection of cervix with acetic acid and hematuria from surveys. We aimed to derive a useful risk score for FGS. The derivation cohort included 349 participants. We used 5-fold iterative resampling for cross-validation. The risk score was tested in a holdout set of 150 participants. The tool attained a sensitivity of 77% to detect FGS; however, the generalizability of this simple and cost-effective tool is yet to be explored. Our current study builds on this by using advanced AI methods to directly analyze colposcopy images for FGS lesions. This approach aims to provide an accurate, objective diagnosis that does not rely on symptoms alone or on limited expert availability. In this way, the previous tool helped to highlight the need for better diagnostics, and our AI model is a step forward in meeting that need. Together, these efforts are part of a larger goal to improve detection and treatment of FGS, especially in areas with limited healthcare resources.

Studies of computer-aided FGS lesion diagnosis are sparse. One that has been published includes colorimetric analyses of 30 cervical images with yellow sandy patches that were diagnosed by a clinician during colposcopy and achieved a sensitivity of 83% [12]. Subsequent colorimetric analysis of yellow sandy patches by the same research group using almost 700 cases had a sensitivity of 80.5% [13]. This same research group also used morphological analyses of 150 images to identify abnormal blood vessels indicative of FGS with a sensitivity of 78% [14]. These studies were focused on just two of the four indicators of FGS (yellow sandy patches and abnormal blood vessels), and none used modern AI methods, which leaves room for the development of new AI-based FGS-specific diagnostic algorithms. Another research study [15] focused on creating and validating a measure called cervical lesion proportion (CLP) to quantify cervical pathology in FGS. Researchers used a digital imaging technique, overlaying a grid with 424 identical squares on high-resolution images of the cervix from 70 women with FGS. In a similar paradigm, rubbery papule count (RPC) was also computed. The intraclass correlation coefficient for CLP and RPC were 0.94 and 0.88 for inter-rater reliability and 0.90 and 0.80 for intra-rater reliability. The more recent work by Zhu et al. [16] used advanced deep learning model, You Only Look Once (YOLO) to diagnose FGS on 125 subjects with the model trained on 504 subjects. The study performed detection and localization of four key FGS indicators. For each subject 2 images were used and final prediction was made using voting. The model attained sensitivity of 96% and accuracy of 78%. YOLO models have shown their success in another related study by Maturana et al [47] for identifying the Schistosoma haematobium eggs in urine samples obtained from microscopy images. In summary, our study emphasizes robust preprocessing and harmonization, including specular-reflection removal and region-of-interest cropping, which allow the model to extract diagnostic features even from low-quality, variable-illumination images and improve generalizability. However, we acknowledge that external validation has not yet been performed. Our model was developed and tested exclusively on images acquired from Bovie Colpo-Master colposcopes at two Zambian study sites, and we cannot make claims about generalizability to other colposcope devices, imaging conditions, or populations. In future, external validation across different colposcope brands, clinical settings, and endemic regions is essential. We agree that the dataset size and imbalance represent substantive limitation in our study. With 340 subjects and only 92 FGS-positive cases, the dataset may not fully capture the heterogeneity of FGS presentations across different populations, However, the limited number of FGS-positive subjects reflects the rarity of clinically confirmed cases and the challenges of diagnosis and data collection in endemic regions. This constraint likely impacts generalization and contributes to model variance. Hence, in future there is a need for multi-center data collection efforts to build larger, more diverse training cohorts. Such expansion would not only improve statistical power but also enhance model robustness across clinical settings, an essential requirement for scalable, real-world deployment.

The importance of high sensitivity to avoid missing FGS cases is well known, especially because untreated infections can have serious health effects such as kidney damage and fibrosis of the bladder and ureter in advanced cases [2]. However, a certain number of false positive results can cause problems. A subject misdiagnosed as FGS positive can face social stigma in many communities [10]. This stigma can cause discrimination, problems in relationships, and emotional stress for the women affected [10]. It is reported that fear of stigmatisation might hinder women to disclose FGS-associated symptoms [10]. Chronic FGS lesions can be prevented by regular treatment with praziquantel when started at an early age and continued throughout life. But it is not always available in all areas where FGS is common, and shortages have been reported in some places [55]. Using praziquantel for people who do not actually have FGS can put pressure on limited healthcare resources. Praziquantel resistance has not been documented in S. haematobium populations to date thus judicious use of the drug is recommended to preserve its efficacy. Thus, while it is important to catch as many true cases as possible, steps to improve the accuracy of diagnosis and confirm positive results can help reduce negative effects on women, healthcare systems, and drug use.

FGS lesions may be more severe with repeated exposures, potentially reducing the effectiveness of treatment [18]. In the future, an AI tool could be useful not only for diagnosis but also for treatment effect monitoring, especially in women with more severe lesions and in rural endemic areas where repeated exposure is high [1]. Further, in the future, it may be possible to develop a smartphone-based app that could be deployed in clinical settings to diagnose FGS by frontline healthcare workers. Moreover, integrating healthcare workers into AI-assisted diagnostic workflows, as demonstrated in recent studies [16], could enhance training and facilitate broader adoption of FGS diagnostic tools in resource-limited settings. Importantly, such an AI tool has the potential to support point-of-care diagnosis and monitoring using low-cost imaging devices, even in remote areas without access to expert clinicians or colposcopes. With appropriate validation and integration into existing strategies, AI-assisted diagnosis could help guide timely treatment with praziquantel, support follow-up after drug administration, and ultimately contribute to reducing the long-term burden of FGS in endemic communities.

Some limitations in our study warrant consideration. A significant limitation of our approach is that the algorithm focuses primarily on the cervix and transformation zone, with exclusion of the vaginal walls and fornices. FGS is diagnosed by visual inspection of characteristic lesions on the cervix and vaginal wall [9]. Women with vaginal wall lesions alone may not be detected by our current model because vaginal walls are very difficult to consistently visualize and photograph during colposcopy [56]. Anterior and posterior surfaces of the vaginal wall can be inspected by rotating the speculum 90 degrees [57]. Hence, a single image focused on cervix cannot capture vaginal walls. In order, to include analysis of vaginal walls, multiple images need to be captured. Clinicians may easily miss the localized and faint signs of *S. haematobium* infection within the female lower genital tract [56]. Similarly, FGS may involve the upper reproductive tract, the structures of which are not visualized at all during colposcopy [56]. These are known limitations in the field of FGS diagnosis. Future algorithm development should include additional images of vagina and methods to systematically include and analyze vaginal wall regions. Additionally, complementary diagnostic approaches such as pelvic imaging or histopathological assessment can be done to capture FGS manifestations in less accessible areas of the reproductive tract in future studies to establish more accurate ground truths. Our cohort consisted of sex workers and single mothers; it will be important to analyse whether findings from these subgroups of women are generalizable to the general population. Another limitation was that women positive for FGS with concurrent STIs were not excluded from the analyses. Thus, we were unable to determine if the presence of another infection affected the sensitivity/specificity of the AID-FGS. Trichomoniasis has a weak association with FGS [58].

## Conclusions

Our study on the ability of various deep learning models and handcrafted features for diagnosing FGS using images of the cervix taken during colposcopy indicated that the Ensembled model performed better than other counterparts as well as the handcrafted features. The use of AI/machine learning models for identifying FGS from cervical photographs could significantly enhance early diagnosis and treatment, potentially reducing the morbidity associated with FGS and lowering the risk of HIV transmission in affected populations.

## Supporting information

S1 Text This document provides a comprehensive description of the model training setup. It also includes the distribution of severity scores in the test dataset. (DOCX)
