# Supplementary material for: AID-FGS: Artificial intelligence-enabled diagnosis of female genital schistosomiasis: Preliminary findings
Source: PLOS Digit Health. 2026 Feb 20;5(2):e0001255. doi: 10.1371/journal.pdig.0001255 (PMC12923067; doi:10.1371/journal.pdig.0001255)
Supplement: S1 Text — (DOCX) [file pdig.0001255.s001.docx]

1. **Transfer Learning:** As we move from the initial layers of a trained Convolutional Neural Network (CNN) to the deeper layers, the features transition from generic to more task specific. Therefore, for fine-tuning on a target domain, the deeper pre-trained layers can be substituted with new, randomly initialized layers and retrained using the target data. In our study, we appended the dense layers (256 neurons) followed by *ReLu, BatchNorm1d, Dropout and Linear layer* (2 neurons) to the fourth layer from the end. The pretrained weights of each model were not updated during fine-tuning. The activations of all three models from the last Linear layer were averaged to get the final predictions scores.
2. **Model Training:**

The S_t_ was augmented using transforms listed in Additional Table 1 to make the model generalize better to new, unseen data. The augmentations were done with respect to flipping the images, change in brightness, hue and saturation of images. Other augmentation parameters were related to rotation, blurring, improve the contrast of images by adaptive histogram equalization. The parameters used for training are listed in Additional Table 2. The model was trained for 200 epochs/iterations by penalizing the loss function for misclassified FGS samples.

**Table A: List of Augmentation parameters**

| **Transform** | **Parameters** |
| --- | --- |
| Resize | (224,224) |
| Horizontal Flip, Vertical Flip | P=0.5, p=0.5 |
| Random Brightness Contrast | P=0.2 |
| Hue Saturation | P=0.2 |
| Coarse Dropout | P=0.5, max holes=1, max height=50, max Width=50, min holes=1, min height=50, min width=50 |
| Rotate | Limit=30, p=0.5 |
| Shift Scale Rotate | Shift limit=0.1, scale limit=0.1, rotate limit=30, p=0.5 |
| Grid Distortion | P=0.5 |
| Gaussian Blur | Blur limit= (3,7), p=0.5 |
| Contrast-limited adaptive histogram equalization | P=0.5 |
| RGB Shift | P=0.5 |

**Table B: List of training parameters**

| **Training parameters** |
| --- |
| Epochs=200 |
| Loss function: Weighted Cross Entropy Loss [04,0.6] |
| Optimizer: Stochastic Gradient Descent |
| Momentum=0.9 |
| Weight Decay=2e-3 |
| Learning rate=0.00002 |
| Batch size=16 |

**Fig A**

With only 21 FGS-positive subjects in the test set, analyzing 8 individual severity categories would result in very small sample size. Figure below shows histogram of scores in the test set. For example, only one subject in the test set has score 7. The dichotomization into "lower severity" (scores 1-4) and "higher severity" (scores 5-8) reflect a clinically meaningful distinction and helps to gain some intuition from data


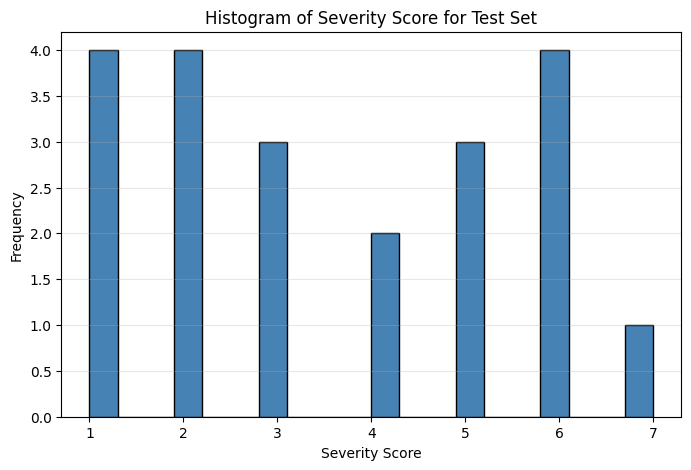
 **Fig A:** Distribution of FGS-positive subjects across the 8-point severity scale in the test set (N=21).
